# Innate Immune Response to Influenza Virus at Single-Cell Resolution in Human Epithelial Cells Revealed Paracrine Induction of Interferon Lambda 1

**DOI:** 10.1128/JVI.00559-19

**Published:** 2019-09-30

**Authors:** Irene Ramos, Gregory Smith, Frederique Ruf-Zamojski, Carles Martínez-Romero, Miguel Fribourg, Edwin A. Carbajal, Boris M. Hartmann, Venugopalan D. Nair, Nada Marjanovic, Paula L. Monteagudo, Veronica A. DeJesus, Tinaye Mutetwa, Michel Zamojski, Gene S. Tan, Ciriyam Jayaprakash, Elena Zaslavsky, Randy A. Albrecht, Stuart C. Sealfon, Adolfo García-Sastre, Ana Fernandez-Sesma

**Affiliations:** a Department of Microbiology, Icahn School of Medicine at Mount Sinai, New York, New York, USA; b Department of Neurology, Center for Advanced Research on Diagnostic Assays, Icahn School of Medicine at Mount Sinai, New York, New York, USA; c Department of Medicine, Icahn School of Medicine at Mount Sinai, New York, New York, USA; d The Graduate School of Biomedical Sciences, Icahn School of Medicine at Mount Sinai, New York, New York, USA; e Global Health and Emerging Pathogens Institute, Icahn School of Medicine at Mount Sinai, New York, New York, USA; f Infectious Diseases, J. Craig Venter Institute, La Jolla, California, USA; g Department of Medicine, University of California San Diego, La Jolla, California, USA; h Department of Physics, The Ohio State University, Columbus, Ohio, USA; Hudson Institute of Medical Research

**Keywords:** epithelial cells, influenza, innate immunity, interferons, single cell

## Abstract

Early interactions of influenza A virus (IAV) with respiratory epithelium might determine the outcome of infection. The study of global cellular innate immune responses often masks multiple aspects of the mechanisms by which populations of cells work as organized and heterogeneous systems to defeat virus infection, and how the virus counteracts these systems. In this study, we experimentally dissected the dynamics of IAV and human epithelial respiratory cell interaction during early infection at the single-cell level. We found that the number of viruses infecting a cell (multiplicity of infection [MOI]) influences the magnitude of virus antagonism of the host innate antiviral response. Infections performed at high MOIs resulted in increased viral gene expression per cell and stronger antagonist effect than infections at low MOIs. In addition, single-cell patterns of expression of interferons (IFN) and IFN-stimulated genes (ISGs) provided important insights into the contributions of the infected and bystander cells to the innate immune responses during infection. Specifically, the expression of multiple ISGs was lower in infected than in bystander cells. In contrast with other IFNs, IFN lambda 1 (IFNL1) showed a widespread pattern of expression, suggesting a different cell-to-cell propagation mechanism more reliant on paracrine signaling. Finally, we measured the dynamics of the antiviral response in primary human epithelial cells, which highlighted the importance of early innate immune responses at inhibiting virus spread.

**IMPORTANCE** Influenza A virus (IAV) is a respiratory pathogen of high importance to public health. Annual epidemics of seasonal IAV infections in humans are a significant public health and economic burden. IAV also causes sporadic pandemics, which can have devastating effects. The main target cells for IAV replication are epithelial cells in the respiratory epithelium. The cellular innate immune responses induced in these cells upon infection are critical for defense against the virus, and therefore, it is important to understand the complex interactions between the virus and the host cells. In this study, we investigated the innate immune response to IAV in the respiratory epithelium at the single-cell level, providing a better understanding on how a population of epithelial cells functions as a complex system to orchestrate the response to virus infection and how the virus counteracts this system.

## Abstract (precis)

Influenza A virus (IAV) is a respiratory pathogen of high importance to public health. Annual epidemics of seasonal IAV infections in humans are a significant public health and economic burden. IAV also causes sporadic pandemics, which can have devastating effects. The main target cells for IAV replication are epithelial cells in the respiratory epithelium. The cellular innate immune responses induced in these cells upon infection are critical for defense against the virus, and therefore, it is important to understand the complex interactions between the virus and the host cells. In this study, we investigated the innate immune response to IAV in the respiratory epithelium at the single-cell level, providing a better understanding on how a population of epithelial cells functions as a complex system to orchestrate the response to virus infection and how the virus counteracts this system.

## INTRODUCTION

The innate immune response is the first host defense against virus infection. Influenza A virus (IAV) targets epithelial cells in the respiratory tract for replication. Therefore, it is critical to understand the innate immune mechanisms that protect these cells from infection by IAV. In airway epithelial cells, IAV double-stranded and triphosphorylated RNAs are pathogen associated molecular patterns (PAMPs) detected by receptors such as retinoic acid-inducible gene I (RIGI), melanoma differentiation-associated protein 5 (MDA5), and Toll-like receptor (TLR) 3 (3–7). Upon PAMP detection, several autocrine signaling pathways are initiated, leading to expression of type I and III interferons (IFNs) and other cytokines (8, 9). Engagement of type I and type III IFN to their receptors, IFN alpha/beta receptor (IFNAR1-IFNAR2) or IFN lambda receptor 1 (IFNLR1-IL10R2), respectively, results in a paracrine response and subsequent activation of hundreds of IFN-stimulated genes (ISGs), which have antiviral activity and therefore will protect neighboring cells from viral infection (10). Signaling events downstream of IFNAR1-IFNAR2 and IFNLR1-IL10R2 are highly similar, despite the sequence and structure of these receptors being extremely different (11). These events include activation of Janus kinase (JAK) family kinases, phosphorylation and dimerization of the signal transducer and activator of transcription 1 (STAT1) and STAT2, association between STAT complexes and interferon responsive factor 9 (IRF9) to form interferon-stimulated gene factor 3 (ISGF3), and translocation of ISGF3 to the nucleus, where it binds to specific DNA elements to induce ISG expression (12). Nevertheless, one important difference which confers distinct functions to the two receptors is their cellular and tissue distributions. While type III IFN signaling is particularly important in the epithelial surfaces of the respiratory tract, gastrointestinal tract, skin, liver, or the lining of the blood-brain barrier (13), type I IFN receptor expression in tissues is ubiquitous. In particular, expression of IFNLR1-IL10R2 by respiratory epithelial cells has been shown to be important for antiviral immunity against IAV *in vivo* and in cell culture (14–17).

Most of the viruses that infect humans have developed strategies to counteract the innate immune system by diverse mechanisms. One of the best-characterized examples is mediated by IAV nonstructural protein 1 (NS1). It is known that NS1 inhibits the detection of viral RNA by interacting with RIGI (18) and with the ubiquitin ligases TRIM25 (19) and RIPLET (20), which leads to reduced IRF3 and NF-κB activation and decreased type I IFN production. NS1 also binds directly to double-stranded RNA (dsRNA) and sequesters it, preventing recognition and activation of the 2′,5′-oligo(A) synthetase (OAS)-RNase L pathway (21) and the type I IFN-induced protein kinase RNA activated (PKR) (22, 23, 24). NS1 has also been shown to counteract immune cellular responses by interacting with the RNA posttranscriptional processing machinery (25–28) and to promote translation of viral mRNA (29–32). NS1 is involved in the regulation of phosphatidylinositol 3-kinase (PI3K) activation by binding to the p85β subunit (33, 34). Additionally, the C-terminal tail of H3N2 NS1 was found to act as a histone tail mimic and reduce host transcription (35). Other viral components in addition to NS1 may also contribute to viral immune antagonism. For example, the hemagglutinin (HA)-encoding segment of pandemic IAV has been reported to suppress immunogenic cell death (36). The PB1-F2 and PB2 viral proteins have been shown to prevent mitochondrial antiviral-signaling protein (MAVS) activation and IFN induction (37, 38), and the PA-X protein has been reported to degrade cellular mRNA (46).

Therefore, there is a complex interplay between the innate immune responses elicited in the cell and how the virus counteracts this cellular response. Many aspects of the dynamics of the complex interactions that occur after IAV infection are not well understood. In order to better understand these dynamics, we characterized expression patterns of host and viral factors during IAV infection at the single-cell level. For these studies we used human respiratory cells infected with IAV at a low or high multiplicity of infection (MOI). First, time-lapse microscopy experiments showed MOI-dependent expression of NS1 per cell. Next, the single-cell transcriptome analysis also showed MOI-dependent expression of viral genes, with a negative correlation of the levels of the viral NS1 and PA genes with cellular gene expression, and distinct patterns of type I and III IFN and ISG expression in infected versus bystander cells. In addition, we found that type III IFN responses have a component of paracrine induction in epithelial cells. These findings were possible thanks to the use of single-cell transcriptome analysis, which, in contrast to bulk analysis, allowed for the characterization of gene expression profiles in infected versus bystander cells, as well as a stratification of the cellular gene expression according to the levels of the expression of viral genes. Finally, we determined the time scales of the functional antiviral responses during the first two rounds of IAV infection using a staggered IAV H1N1/H3N2 coinfection model in normal human bronchial epithelial (NHBE) cells.

## RESULTS

### Temporal dynamics of NS1 IAV expression in epithelial cells.

**FIG 1:**
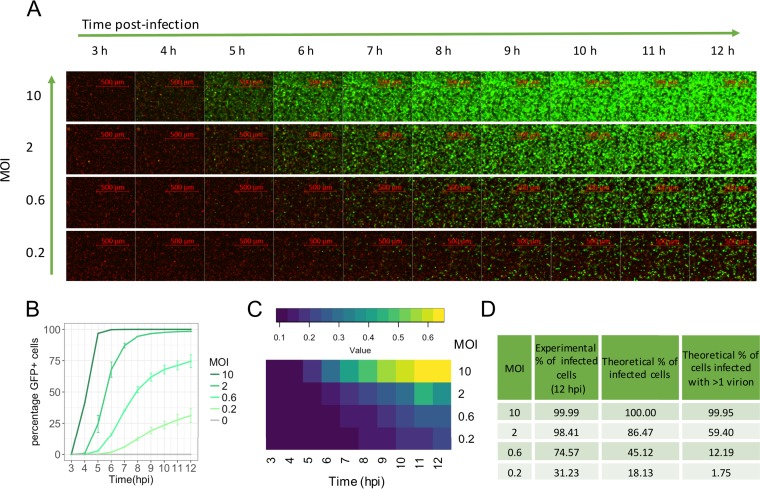
MOI-dependent temporal expression dynamics of the NS1 protein (PR8-GFP virus) in human respiratory epithelial cells (A549 H2B-mCherry) using time-lapse microscopy. Green indicates NS1 expression (GFP). The cell nuclei (H2B-mcherry) are depicted in red. (A) Time course from 3 to 12 hpi. One representative field is shown for each MOI. (B) Quantification of the number of GFP-positive cells for the four MOIs used (approximately 25,000 cells/well). For MOI of 0.2, 0.6, and 2, averages of two replicate wells ± SDs are shown. For infection at an MOI of 10 and mock infection, data from one single well are shown. (C) Heat map showing the median of fluorescence intensity of the infected cells (GFP+ cells). (D) Table showing the measured percentages of infected cells at 12 hpi, as well as the theoretical percentages of cells infected either with ≥1 virion (all infected cells) or >1 virion (multiple infections).

The IAV NS1 protein is one of the viral antagonists of host innate immune responses. We first investigated the time scales of NS1 expression during IAV infection by time-lapse microscopy in the A549 human epithelial cell line using a green fluorescent protein (GFP)-tagged NS1-expressing virus (PR8-GFP) (40). A549-H2B-mCherry was used to improve nucleus image segmentation accuracy in live imaging. We infected cells at MOIs of 10, 2, 0.6, and 0.2 and captured images from 3 to 12 h postinfection (hpi), which would mainly correspond to a single cycle of replication (41). As shown in Fig. 1A, with an MOI of 10, cells expressing NS1 were detected as early as 4 hpi. However, as the MOI was reduced, we observed a delay in the detection of expression of NS1, with detection at 5 hpi at an MOI of 2, 6 hpi at an MOI of 0.6, and 7 hpi at an MOI of 0.2. Quantification of the percentage of infected cells in the time-lapse experiment is shown in Fig. 1B. When we measured the median fluorescence intensity per cell in the infected cells (GFP+ cells), we found that a higher MOI was associated with higher levels of NS1 expression per cell at every time point (Fig. 1C). This phenomenon can be explained by the differences in the number of cells infected with multiple infectious viruses, which will decrease with the MOI. According to the Poisson distribution [P(k) = e^−MOI^(moi^k^/k!), where k is number of virus particles that infects each cell and P(k) the fraction of cells that receive k particles], in a cell culture exposed to an MOI of 10 virtually all cells would theoretically receive two or more virions, and this number is reduced as the MOI diminishes to less than 2% with an MOI of 0.2 (Fig. 1D). Thus, both earlier and higher NS1 expressions per cell were associated with increasing proportions of cells infected by more than one virus.

### Single-cell analysis of the innate immune response and viral antagonism during IAV infection in respiratory epithelial cells.

**FIG 2:**
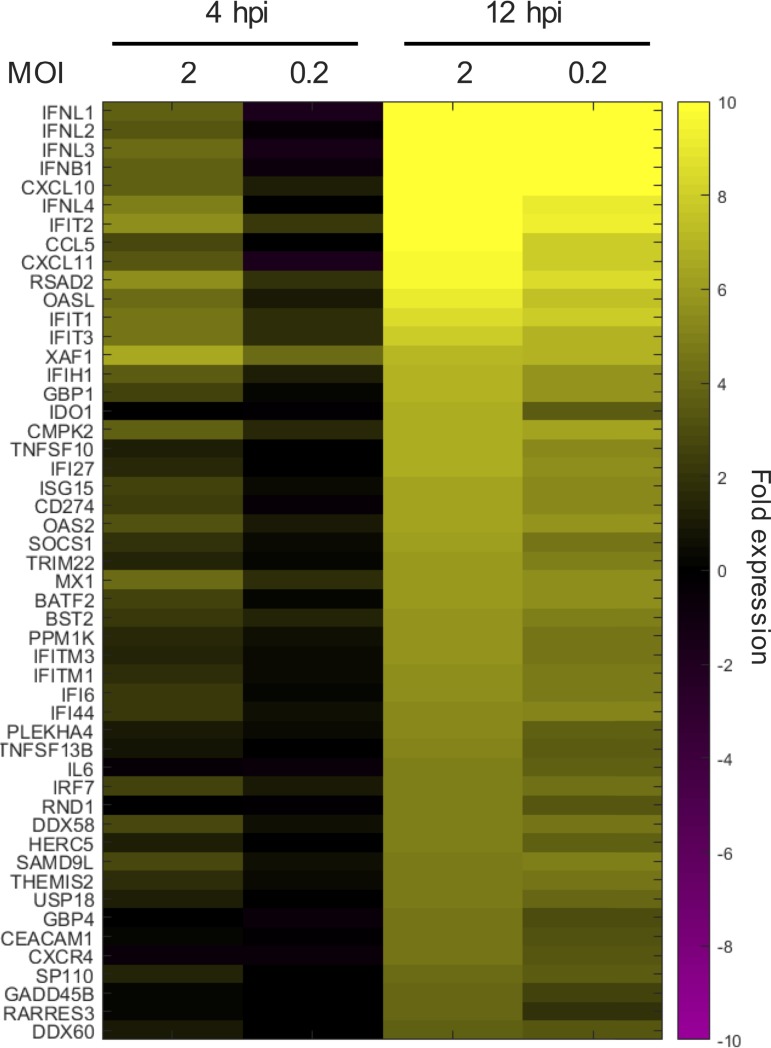
Single-cell RNA transcriptome analysis of PR8-GFP-infected epithelial cells. A heat map shows the expression levels of the 50 markers with the highest upregulation in infected versus mock-infected cultures (mean UMI count for each sample compared to value for the time-matched mock infection).

Based on the results from our time-lapse microscopy experiments, we hypothesized that the MOI dependence of NS1 expression could have important implications for its antagonism of the innate immune responses. Therefore, we performed single-cell RNA expression profiling (10× Genomics) in the same A549/PR8-GFP system using an MOI of 2, at which around 59% of the cells should be subjected to infections with more than one virus, and an MOI of 0.2, which should result in a low percentage of cells infected by more than one virus (∼2%). First, we identified the genes that showed the largest fold increase in mean expression (normalized unique molecular identifier [UMI] counts) across all cells in the infected versus the mock-infected cultures (Fig. 2). None of the IFNA genes were among the highly upregulated genes, indicating low levels of induction. As expected, most of the identified upregulated genes were cytokine and chemokine genes and ISGs.

**FIG 3:**
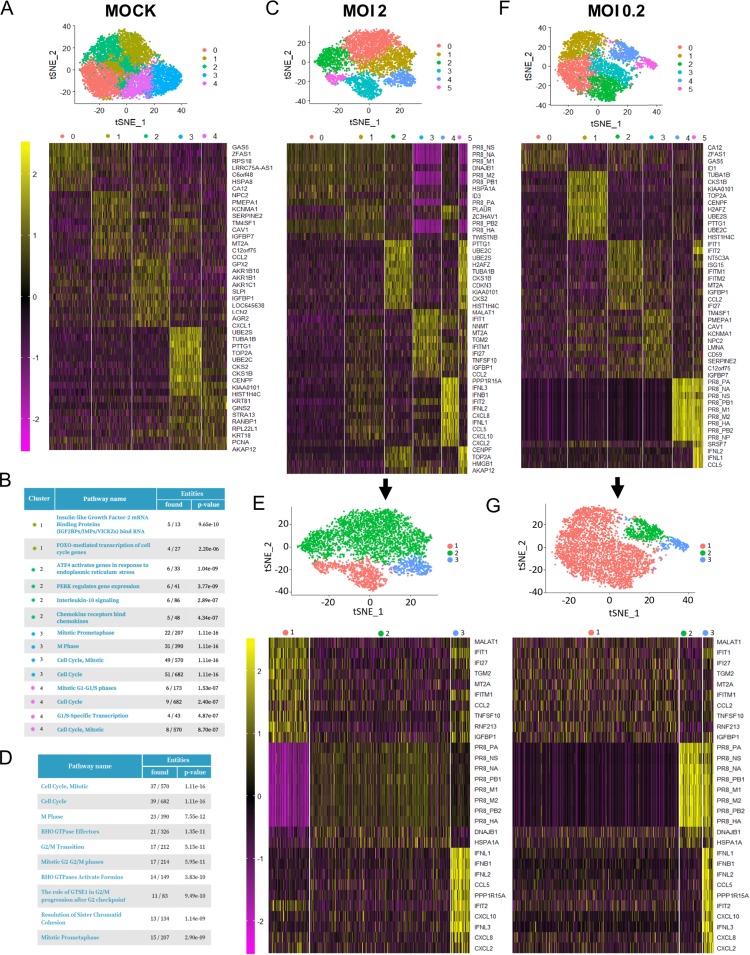
tSNE visualization, unsupervised clustering analysis, and heat maps showing the most differentially expressed genes identified by single-cell RNA sequencing in mock-infected cultures (A) and infected cultures (C, E, F, and G) at 12 hpi. This clustering strategy resulted in 5 clusters in the mock-infected cultures (A), mostly due to differences in genes associated with cell cycle as shown by a Reactome pathway Database analysis (42) (B). The most represented pathways for each cluster (up to 4 per cluster) are depicted (*P* < 0.001; cluster 0 analysis did not yield any significant pathway). Some of these genes also mediated clustering in infected cultures, resulting in 6 different clusters at MOIs of 2 and 0.2 (C and F). (D) The 10 most relevant pathways (sorted by *P* value) identified by a Reactome pathway Database analysis of the 111 genes or entities that were differentially expressed between cluster 2 and clusters 0, 1, and 4 of the sample infected at an MOI of 2. (E and G) The three clusters generated after manual curation of the samples infected at MOIs of 2 and 0.2: infected cells producing high levels of IFN (blue), infected cells producing low levels of IFN (green), and mostly uninfected cells (red). The scale of the heat maps shows the gene expression of each gene in each cell relative to the mean expression of that gene across the sample (note that expression levels between different samples cannot be compared, as they are normalized across the cells in each sample).

*t*-Distributed stochastic neighbor embedding (tSNE) visualization of the single-cell data, followed by a clustering analysis (see Materials and Methods), was used to examine the global expression profile of the single cells (Fig. 3). At 12 hpi, for each MOI, we initially identified five clusters in the mock-infected cultures (Fig. 3A) and six clusters in the IAV-infected samples (Fig. 3C and F). However, by comparing the differentially expressed genes among the 5 clusters in mock-infected cultures and subsequent pathway analysis using a Reactome Pathway Database tool (42) (Fig. 3B), we found that this phenomenon was mostly due to genes involved in cell cycle. Part of the clustering identified in the mock-infected cultures also mediated the clustering in the infected cultures (heat maps in Fig. 3C and F). For instance, at an MOI of 2, cluster 2 (Fig. 3C) seemed to show a clear separation from clusters 0, 1, and 4 and had 111 genes that were differentially expressed than in those clusters. A pathway analysis of those genes (42) identified that the main pathways represented were associated with cell cycle processes (Fig. 3D). Therefore, in order to extract the information relevant to this study, we collapsed some of these clusters according to the expression of viral genes and genes associated with the innate immune response. As a result, we defined three new clusters for each MOI as depicted in Fig. 3E and G. For both MOIs, the red cluster is composed mostly of cells showing undetectable or low levels of viral mRNA expression. The green and blue clusters showed expression of viral genes; therefore, they are composed mostly of infected cells. Cells in the blue cluster (also for both MOIs) showed higher expression of type I and type III IFNs as well as chemokines such as CCL5, CXCL10, and CXCL8 (Fig. 3E and G, bottom sections of the heat maps) than the green cluster and, as expected, than the red cluster (which is mostly composed of bystander cells). Interestingly, at an MOI of 2, cells belonging to the blue cluster clearly showed lower expression of viral genes, suggesting that they might experience a lower antagonist effect leading to a higher IFN production (Fig. 3E). Another interesting observation was that the red cluster showed overall higher expression of ISGs such as IFIT1, IFI27, and IFITM1 (Fig. 3E, top section of the left heat map) than the infected cells. A similar pattern was found at an MOI of 0.2. However, at this MOI we did not find clear differences in the expression of viral genes between the blue (cells producing high levels of IFN) and the green clusters (Fig. 3F). Cells infected for 4 h showed profiles very similar to those of mock-infected cells, with the exception of the presence of low numbers of infected cells and few innate immune genes as differentially expressed genes at an MOI of 2 (data not shown). Therefore, we focused most of our subsequent analysis on the 12-h time point.

**FIG 4:**
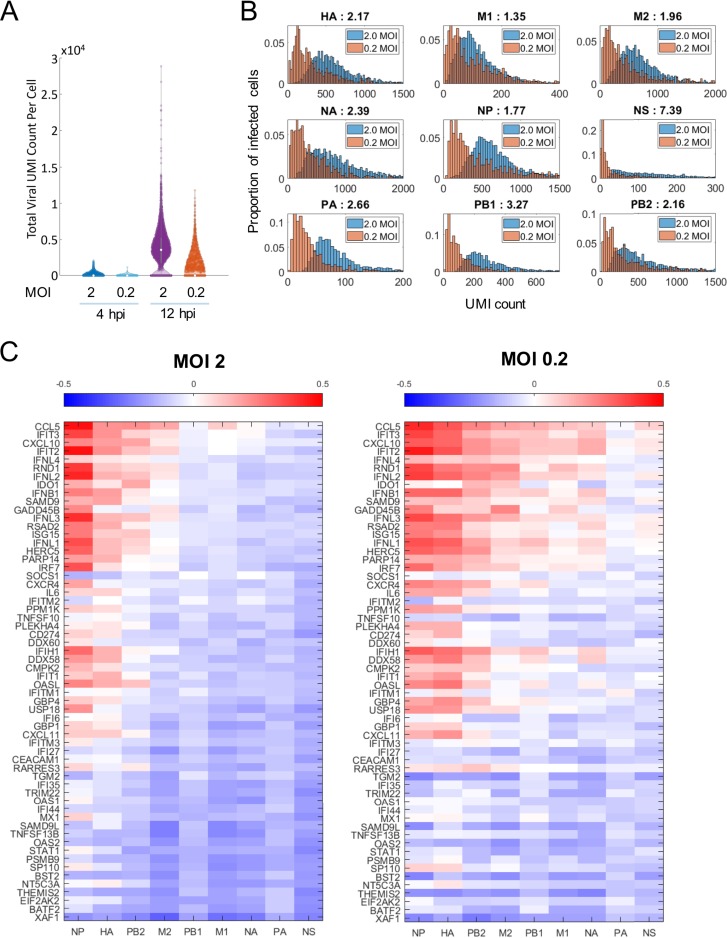
Distribution of the expression of viral genes (A and B) and their correlation with cellular genes (C). (A) Total viral UMI counts per cell. (B) Cells infected with a higher MOI (i.e., 2) show more elevated levels of viral genes than those infected with a lower MOI (0.2). The ratio of the median UMI count per cell for each MOI is shown at the top of each plot. (C) Heat map showing the correlation (Pearson correlation coefficient) between viral genes and a comprehensive panel of cellular genes. Viral and cellular genes were sorted by their median correlation coefficients against the respective gene sets.

Given the distinct patterns of gene expression of cellular and viral genes among the different groups, we tested whether there was an MOI-dependent expression pattern of all viral genes as we found for NS1 by time-lapse microscopy. Consistent with those findings, we found that at an MOI of 2, there was a higher level of expression of total viral RNAs per cell than at an MOI of 0.2 (Fig. 4A). At 12 h, there was a clear gap in the detection of viral RNAs between bystander cells (0 viral UMI) and infected cells (over 648 and 349 at 2 and 0.2 MOI, respectively), which allowed for better differentiation between these two groups in the culture in subsequent analyses. Approximately 86% and 20% of infected cells were identified at MOIs of 2 and 0.2, respectively, at 12 hpi. Next, we analyzed the levels of the different IAV genes per infected cell. As shown in Fig. 4B, cells infected with a high MOI contained more viral UMIs/cell that those infected with a low MOI, supporting the hypothesis that multiple infections per cell result in increased level of viral proteins. Interestingly, and in accordance with previous reports (43–45), we found great heterogeneity in the numbers of viral transcripts across the cells in infected cultures for both MOIs.

Subsequently, we investigated the implications of the MOI-dependent expression of viral genes in the innate immune response elicited in infected cultures. We analyzed the correlation between the viral and cellular transcripts that we found to be upregulated during IAV infection. As shown in Fig. 4C, we observed that at the high MOI, most of the viral genes had a negative correlation with cellular genes, with the exception of the NP and HA genes. At an MOI of 0.2, only the NS and PA genes showed a negative correlation with most of the cellular genes. In both cases, the viral genes that showed the strongest negative correlation with cellular genes were the NS and PA genes, supporting the conclusion that viral proteins encoded by these genes might have an important function as negative regulators of transcription of cellular genes. This effect is more evident with an elevated MOI, due to high levels of expression of viral genes per infected cell. Interestingly, the NS gene encodes the known innate immune antagonist NS1 protein, while the PA gene encodes the PA protein, which is part of the polymerase complex, and the PA-X protein and is also known to repress cellular gene expression (46, 47). In conclusion, we found that the expression of viral proteins per cell is MOI dependent—due to infection by multiple viruses per cell at the higher MOI—and is associated with reduced transcription of cellular innate immune genes.

### Infected and bystander cells show differential host gene expression profiles.

**FIG 5:**
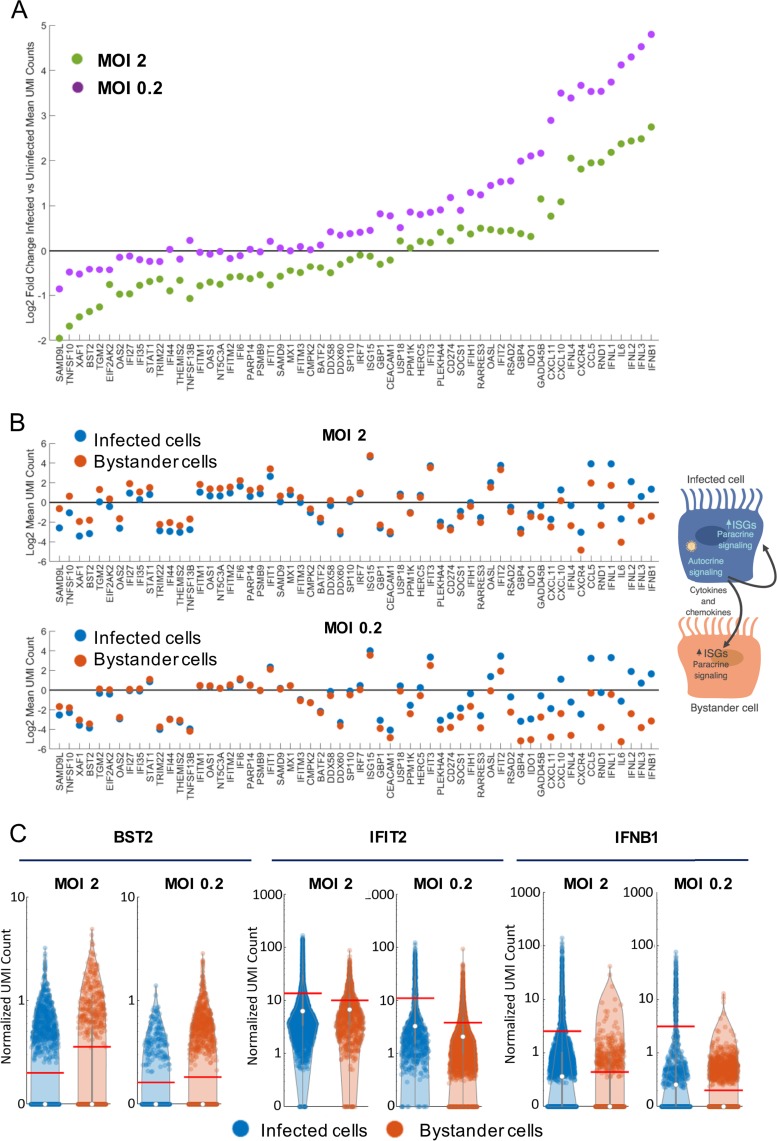
(A) Ratio of the log_2_ fold change mean expression in infected and bystander cells. Genes are sorted by the average value of the log_2_ fold change of mean expression in infected and bystander cells for the two MOIs. (B) Comparison of the mean UMI count (log_2_) of cellular genes in infected and bystander cells for MOIs of 2 and 0.2. Data above the horizontal line indicate increased expression in infected versus uninfected cells. Data below the horizontal line indicate decreased expression in infected versus uninfected cells. (C) Violin plots of normalized UMI count for selected genes at 12 hpi in infected and bystander cell populations at high and low MOIs. Each dot represents a cell. The red line reflects the mean value of normalized UMI count across a cell population. The vertical gray line is a box-and-whisker plot representing the median (white circles), the 25th and 75th percentiles (ends of thicker gray lines), and high and low whiskers at 1.5 times the interquartile range (ends of the thin gray lines). In order to better highlight differences in the cell populations while still including zero values, for the *y* axis, we applied a linear scale for values greater than or equal to 0 and less than 1 and a log_10_ scale for values greater than or equal to 1.

Single-cell transcriptome analysis allows us the unique opportunity to tease out differences in the immune responses of infected cells, mainly elicited by sensing and autocrine signaling, and bystander cells, mainly elicited by paracrine signaling. We compared the distributions of gene expression in infected and bystander cells. The ratio of the expression of the top upregulated genes identified in Fig. 2 between infected and bystander cells was calculated (log_2_ fold change of the mean UMI count per cell in infected over bystander cells) and sorted according to the average of the results for the two MOIs (Fig. 5A). We also selected and included in this panel 10 additional genes that were upregulated over mock-infected cultures and had the lowest ratio between infected and bystander cells (log_2_ fold change of the mean UMI count per cell in infected over bystander cells). There was a consistent pattern of expression across all genes, where a lower ratio between infected and bystander cells was found at the high MOI than at the low MOI. In addition, a gradient in the ratio of the expression of those genes between infected and bystander cells was observed at both MOIs. Visualization of the expression of those genes (log_2_ UMI mean) in infected and bystander cells (Fig. 5B) indicated that this pattern is probably due to a combination of different mechanisms of gene activation and different levels of susceptibility to virus antagonism. Genes depicted toward the left axes of the plot in Fig. 5B showed expression in infected cells that was similar to or lower than in bystander cells, consistent with a paracrine mechanism of activation, as known for most of the ISGs. Frequently, the expression of these genes, such as the BST2 (tetherin), TRIM22, OAS2, IFI27, and IFITM2 genes, was lower in infected cells than in bystander cells, with this effect being more evident at the MOI of 2 (Fig. 5B). As an example, detailed data for BTS2 are shown in Fig. 5C. Genes positioned toward the right axis of these plots had greater expression levels in infected than in bystander cells. Most of these genes are known to be induced in an autocrine manner, such as the genes for type I and III IFNs and chemokines such as CCL5 (48), interleukin 6 (IL-6) (49), and CXCL10 (50, 51). However, we found several ISGs, such as IFIT2, RSAD2 (or viperin), OASL, and IFIH1 (or MDA5), that were expressed at higher levels in infected versus bystander cells, suggesting that in addition to being induced upon IFN signaling, they can also be strongly induced directly upon viral sensing in an autocrine manner. Consistent with this hypothesis, increased expression of these four genes was already detected at 4 hpi at both MOIs (Fig. 2). Detailed data for IFIT2 and IFNB1 are shown in Fig. 5C as a representative example of increased expression in infected versus bystander cells. An autocrine component of activation of these ISGs might be of high importance for early innate immune responses to IAV.

### Early innate immune responses are dominated by type III IFN gene expression.

**FIG 6:**
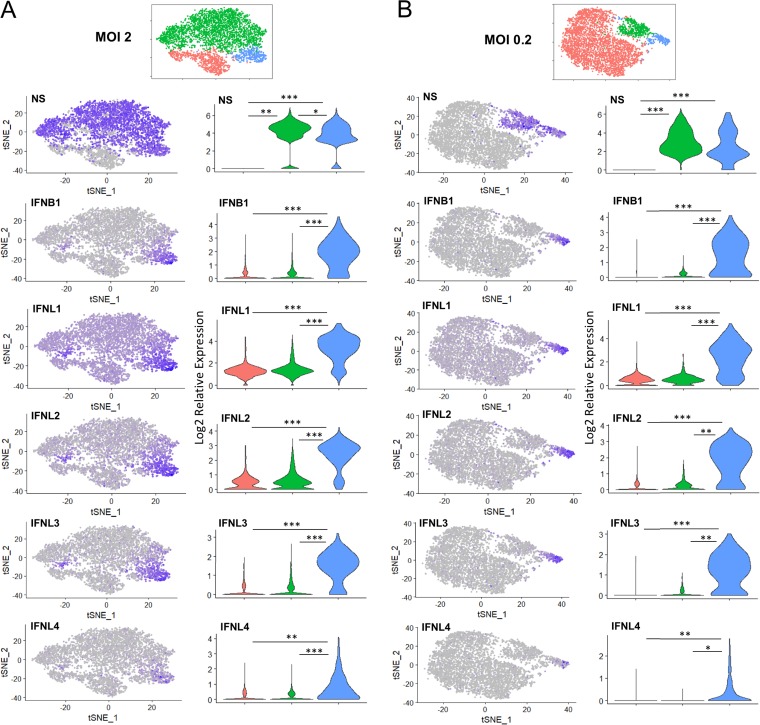
Distribution of IFN expression in infected cultures. Shown are tSNE analysis and violin plots showing the distribution of infected cells (PR8 NS), IFNB1, IFNL1, IFNL2, IFNL3, and IFNL4 at MOIs of 2 (A) and 0.2 (B). IFNL1 shows a broader distribution than the rest of the IFN genes. Levels of expression of NS or IFN are depicted in purple. The density width of the violin shapes indicates the relative frequency of cell expressing specific levels of NS or IFN in each group. A previously described statistical framework for single-cell data that applies a likelihood ratio test was used for comparisons among clusters (95).*, *P* value < 10^−25^ to 10^−100^; **, *P* value 10^−100^ to 10^−200^; ***, *P* value < 10^−200^.

We also analyzed the distribution of type I and type III IFN gene expression in infected cultures at the single-cell level. As can be observed in Fig. 6, we found that the expression of IFNB1 was restricted to a group of infected cells at both MOIs (corresponding to the blue group in the tSNE plots in Fig. 3E and G). A similar pattern was observed for IFNL2, IFNL3, and IFNL4. Interestingly, in the case of IFNL1, while high levels of expression in that specific group of cells were also found, there was a broader pattern of induction of expression among the rest of the cells in the culture, including both infected and bystander cells. At an MOI of 2, there was another small group of infected cells located at the left side of the green cluster (which in general expressed low levels of IFN and chemokines) in the tSNE plots that demonstrated high induction of IFNB1 and IFNL1 to -4. As described above, the preliminary cluster to which these cells belong was formed by the differential expression of genes associated with cell cycle, resulting in a different classification of those few cells than the ones in the blue group (which was characterized by infected cells with high expression of IFN and chemokines).

Quantification of the levels of expression of these type I and III IFN genes and comparison among the three clusters are also shown in Fig. 6. Interestingly, at an MOI of 2, the levels of expression of IAV NS were lower in the blue cluster than in the green cluster, both of them composed mostly of infected cells. The blue cluster again showed elevated levels of expression of all IFN genes, while the cells in the green cluster showed overall levels very similar to those of the red cluster of bystander cells. Consistent with the tSNE visualization, expression of IFNL1 in cells in the red and green clusters was higher than that of IFNB1, IFNL2, IFNL3, or IFNL4. At an MOI of 2, virtually all the cells in the culture expressed IFNL1, albeit to variable levels.

**FIG 7:**
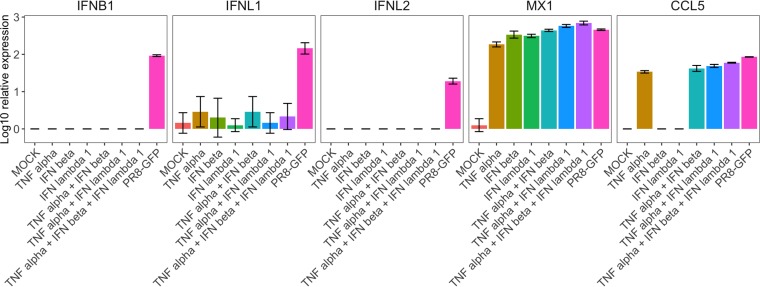
Stimulation of A549 cells with TNF-α, IFN beta, IFNL1 (100 U/ml), or combinations of those cytokines or infected with PR8-GFP (MOI, 2) for 8 h. Induction of IFNL1 was not detected upon stimulation with those cytokines by qRT-PCR. Gene expression of the indicated genes (IFNs, with Mx1 and CCL5 to confirm the effectiveness of the treatments) was analyzed. Averages of biological triplicates ± SDs are shown.

Thus, using single-cell resolution transcriptome analysis, we identified a differential pattern of expression between type I and type III IFN systems during virus infection in epithelial cells. The pattern of IFNL1 expression identified in the single-cell expression analysis was consistent with a paracrine mechanism of activation in a large number of IFNL1-expressing bystander cells, in addition to the well-described autocrine induction upon virus sensing (11). In search of possible inducers of the paracrine expression of IFNL1 induction, we stimulated A549 cells with IFN beta, IFNL1, tumor necrosis factor alpha (TNF-α), or these cytokines combined (Fig. 7). However, we did not detect induction of IFNL1 under any of these conditions, suggesting that other factors released to the medium during infection may be responsible for the broad pattern of expression identified by single-cell analysis.

### Early innate immune responses restrict virus replication in IAV-infected human primary respiratory epithelial cells.

**FIG 8:**
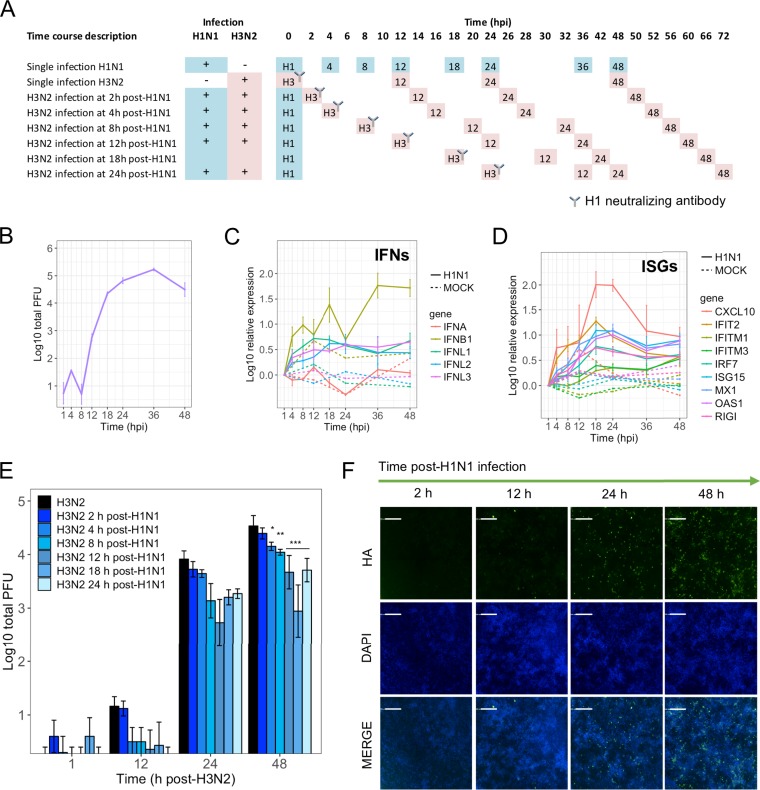
Analysis of the functional antiviral response induced by IAV in differentiated NHBE cells using a H1N1/H3N2 coinfection assay. (A) Scheme of the experimental setup and time line. (B) Replication profile of H1N1 (Cal09) infection of NHBE cells. (C and D) Expression of type I and III IFN and ISGs in NHBE cells in cultures infected with the H1N1 IAV or mock infected. (E) Impact of the innate immune response induced by H1N1 IAV infection on the replication of the H3N2 virus. Averages of biological triplicates ± SEMs are shown. Two-way analysis of variance (ANOVA) and Tukey’s multiple-comparison test were used. Adjusted *P* values are shown as follows: *, <0.05; **, <0.01; and ***, <0.001. (F) Immunofluorescence assay of H1N1-infected NHBE cells, showing a time course analysis of the progression of viral infection.

Our results show that IAV infection at a low MOI results in induction of antiviral ISGs in bystander cells that potentially protect them from viral infection. In order to address the magnitude and timing of the antiviral response induced in bystander cells during IAV infection at a low MOI using primary human respiratory epithelial cells, we established a model of staggered IAV H1N1/H3N2 coinfections in normal human bronchial epithelial (NHBE) cells in an air-liquid interface (1). Using two similar, but distinguishable, viruses to infect cell monolayers at different times allows the analysis of how the antiviral response induced after the first infection affects subsequent infection by the second virus. The design of the experimental protocol is depicted in Fig. 8A. First, we infected NHBE cells with the H1N1 virus A/California/04/2009 at an MOI of 0.05, and then we characterized profiles of gene expression and virus replication throughout a 48-h time course. Using plaque assays (Fig. 8B), we established that the initial release of H1N1 infectious particles was detected at 12 hpi, peaked at 24 to 36 hpi, and declined after 48 hpi. We next analyzed the kinetics of expression of a comprehensive panel of innate immunity-associated genes by quantitative reverse transcriptase PCR (qRT-PCR). Notably, we detected early upregulation of IFNB1, at around 4 hpi, as well as upregulation of IFNL1, IFNL2, and IFNL3, while changes in IFNA expression remained undetected (Fig. 8C). Interestingly, ISGs, including IFIT2, CXCL10, RIGI, and Mx1, were also detected very early after infection (4 to 12 hpi [Fig. 8D]). Therefore, these results demonstrate an early expression of type I and type III IFN and ISGs during IAV infection in epithelial cells.

In the same experiment, additional samples were first infected with H1N1 virus, and then the H3N2 virus A/Wyoming/03/2003 was added at an MOI of 0.05 at selected times points post-H1N1 infection in the presence of the H1 neutralizing antibody 7B2 (Fig. 8A), which is specific for the head region of the A/California/04/2009 isolate and restricts its subsequent rounds of infection. Washes from the apical chamber were collected to assess viral replication at 1, 12, 24, and 48 h after H3N2 infection. The viral titers (PFU) measured after H3N2 infection correspond with H3N2 replication only, since the anti-H1 antibody restricts infections with H1N1 viruses. We used changes in those H3N2 titers as an indicator of the functional antiviral response induced by the previously added H1N1 virus. As both H1N1 and H3N2 viruses were added at a low MOI, numbers of coinfections were negligible. Control experiments demonstrated that the neutralizing antibody 7B2 inhibited H1N1 replication as measured by plaque assay and immunostaining (data not shown). Thus, in this experiment we measured levels of H3N2 replication in bystander cells exposed to paracrine effects from H1N1-infected cells. Analysis of the impact of the innate immune response induced by the initial H1N1 IAV infection on the replication of the subsequently added H3N2 virus showed that adding the H3N2 virus at 2 or 4 hpi resulted in a replication profile very similar to that obtained with the H3N2 single infection. Interestingly, we found a significant decrease in the H3N2 replication titers (48 h post-H3N2 infection) when this virus was added as early as 8 and 12 h post-H1N1 IAV infection. As expected, we observed a greater reduction of the H3N2 IAV replication as the time of addition post-H1N1 IAV infection increased to 24 h or 48 h. To confirm that after infection with the H1N1 virus there was still a sufficiently large number of uninfected cells that could be potentially infected by the H3N2 virus, we performed immunofluorescence staining of NHBE cells infected by H1N1 under the same conditions. Visualization and quantification of complete wells indicated the presence of 1.23% ± 1.32%, 3.2% ± 1.37%, and 6.01% ± 3.00% infected cells at 12, 24, and 48 hpi, respectively (Fig. 8F), indicating that most cells were available for infection upon exposure to the second virus. Therefore, these data indicate that early IFN responses elicited during initial viral infection are effective as early as 8 to 12 hpi and are critical for a functional antiviral response in bystander cells.

## DISCUSSION

The innate immune response is one of the first mechanisms of host defense against viral infections. In order to successfully establish infection in humans, viruses such as IAV have developed strategies to counteract this system. While extensive research on the genes involved in antiviral responses and on IAV antagonism has been performed, the global virus-host interaction and its consequences at the individual cell level are still not well understood. In this study, we performed a deep analysis of these interactions and their functional consequences in respiratory epithelial cells, which are main targets of IAV replication.

**FIG 9:**
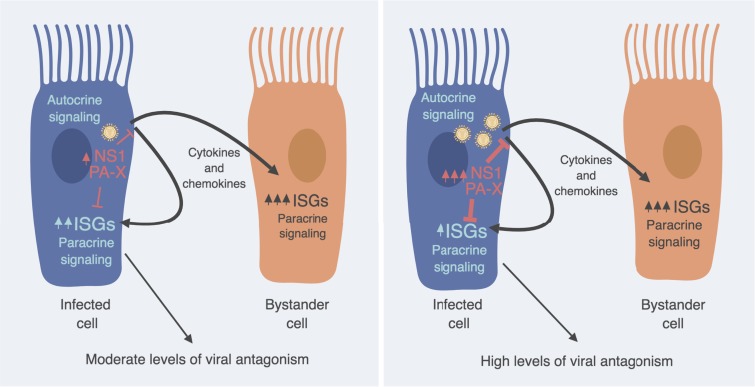
Model of the interplay between IAV and innate immunity in epithelial cells during infection with individual virus particles (left) versus multiple virus particles per cell (right). This illustration was created with BioRender (https://biorender.com).

Our analysis of the dynamics of NS1 expression showed an MOI-dependent expression of this important innate immune antagonist protein in infected cells. Similarly, we found that the levels of all IAV genes per infected cell were more elevated when cells were exposed to a high MOI than to a low MOI, most likely due to differences in the numbers of cells being initially infected with multiple virions. Under physiological conditions, the first round of infection of the respiratory epithelia by IAV would be mediated by a few virus particles, so probably infected host cells receive only one infectious virus. This is consistent with the very low number of viruses shown to be responsible for initation of infection in ferrets (53) and humans (54). However, as infection progresses, infection of neighboring cells is likely mediated by more than one virus originating from the infected cells, with lower numbers of viruses reaching cells farther away from the initially infected cells. Thus, we expect to find both single-virus- and multiple-virus-infected cells during human IAV infections. Interestingly, as found by a correlation analysis of the viral genes versus a panel of innate immune genes, NS shows a consistent negative correlation with the transcription of most of the innate immune genes. This negative correlation is clearly more enhanced in cells infected with multiple viruses (high MOI) than in those infected with single viruses (low MOI), suggesting that the number of virions that infect each individual cell has important consequences for the ability of the virus to counteract the innate immune response in that cell (see model in Fig. 9). Therefore, our results suggest that viral IFN antagonism might be less effective during the initial stages of infection in the context of humans infected with a virus, when cells are impacted by single virus particles. During viral infection, the concentration of viral particles released grows exponentially, reaching levels up to 10^5^ to 10^7^ 50% tissue culture infective doses (TCID_50_)/ml (55, 56). At this level of replication in the respiratory tract tissue, it is highly probable that many cells get infected with multiple viruses, which would enhance the ability of IAV to suppress the innate immune response *in vivo* and at the same time favor the reassortment of IAV genes, an important evolutionary force for IAV.

NS1 has multiple functions to antagonize the cellular response. One of the best characterized is its ability to inhibit virus sensing by RIGI, either by sequestering dsRNA or by direct interaction with RIGI or TRIM25 (19, 57–59). Related to this, we found IFNB1, all type III IFNs, and other cytokines and chemokines among the genes that correlated negatively with the levels of NS at the high MOI. However, we found that gene expression was affected at a general level, suggesting a global inhibition of the transcription of the innate immune genes. Also, when we compared the gene expression profiles in infected versus bystander cells, we found a group of genes, presumably induced in a paracrine manner, that were expressed at lower levels in infected than in bystander cells, probably due to virus transcriptional antagonism. While a combination of viral factors could be responsible for this global shutoff of host transcription, our data suggest that NS1 might be associated with this phenomenon. There is evidence in the literature that IAV infection results in reduced host transcription and mRNA processing by inhibiting the host RNA polymerase II (Pol II) function (60, 61). Related to this, a recent study found that several strains of IAV elicit global deregulation of Poll II transcription termination by impairing 3′-end cleavage and termination, and this effect was dependent on NS1 expression (62). These studies could partially explain the global negative correlation that we found between viral genes and cellular genes. NS1 interactome studies have identified multiple additional interactions of NS1 with nuclear proteins (63, 64), which could also have a global effect on cellular transcription. In addition, another study that used a combination of modeling and experimental research found that ISGs are specifically suppressed in IAV-infected dendritic cells through the inhibition of multiple transcription factors (65). The second viral gene that showed the strongest negative correlation with host genes in our analysis was PA. Interestingly, in addition to encoding one subunit of the IAV polymerase, it has a second open reading frame accessed by ribosomal frameshifting that results in the expression of the protein known as PA-X (46). PA-X inhibits transcription of cellular genes by selectively targeting host Pol II transcribed mRNAs (66). Therefore, it is possible that the global negative correlation is a consequence of the expression of NS1 and PA-X in infected cells. Further investigation will determine if this effect is similar for other IAV strains or if it is strain specific, which will shed light into possible unknown mechanisms for virus-mediated host transcription inhibition.

The interaction between virus infection and the host response was further characterized in our study by comparing the gene expression profiles in infected and in bystander cells. Several genes, including the IFIT2, OASL and RSAD2 (also known as viperin) genes, with known antiviral activity (67–70), and IFIH1 or MDA5, which contributes to viral sensing (71), showed higher expression in infected versus bystander cells, suggesting that these ISGs, in addition to being inducible by type I or III IFN, have also an autocrine component of activation. Indeed, the induction of some ISGs was detected as early as 4 hpi (Fig. 2), with such an early upregulation consistent with direct induction by viral infection. This is in contrast to the highly accepted concept that type I or type III IFN signaling is necessary for the induction of antiviral genes (57, 72), with a few exceptions such as ISG15 and CXCL10 (73–75). The well-described mechanism of activation of transcription of ISGs upon type I/III IFN induction involves assembly and translocation to the nucleus of ISGF3, which binds to the DNA sequences in the promoters of ISGs known as interferon-stimulated response elements (ISRE) (10). Interestingly, Daly and Reich found two complexes in addition to ISGF3, interferon responsive factor 3 (IRF3) and the transcriptional coactivator CREB-binding protein (CBP)/p300, which could recognize ISRE sequences and activate transcription upon dsRNA stimulation in the absence of IFN (73, 76). The abilities of these transcription factors to interact with ISRE differ among various ISGs, due to variations in their DNA sequences. A later study identified IFIT2, ISG15, IFIT3, IFIT1, and GBP1 as IRF3 target genes (77). With the exception of these highly informative studies, literature in this specific topic is scarce. Therefore, direct induction of ISGs in infected cells might be an important player in promoting effective early antiviral responses.

The analysis of the distribution of expression of type I and III IFNs also provided very interesting observations. First, consistent with previous reports (78–80), only a small population of infected cells produced high levels of IFN. NS1 protein could differentially inhibit the IFN response across the infected cells in the culture according to the NS1 intracellular levels, which could be determined by the number of virions infecting the cells. Related to this, at the MOI of 2 we found that the cluster of cells expressing IFN at high levels (Fig. 6A) presented lower levels of the NS gene than the rest of the infected cells. Another possibility is the presence of low levels of defective interfering (DI) particles in the virus stock, which are known to be strong inducers of IFN (81). While virus stocks were carefully prepared to avoid the presence of DI particles, some presence of them cannot be ruled out. However, if a large deletion is present in one segment of a virus, mRNA for the proteins that segment encodes would not be successfully transcribed in the cell infected by that virus. Related to this, we found that at MOIs of 2 and 0.2, 100% and 99.9% of the infected cells, respectively, presented all the different RNA transcripts from the virus, suggesting a low probability for the presence of DI particles in the virus stock. Interestingly, a recent study that used an elegant approach to perform combined cellular and viral single-cell RNA sequencing in IAV-infected A549 cells found that increased proportions of viral defects or mutations, and specifically defects or deletions in NS, were highly associated with increased expression of IFN among infected cells (80).

Our single-cell sequencing data also showed that while the expression of most of the type I or III IFN is restricted to that small cell population, IFNL1 is more widespread, since it is expressed by most of the infected cells and also in the bystander cells, albeit to lower levels. The mechanism for IFNL1 induction in bystander cells is consistent with a paracrine response involving host factors secreted from virus-infected cells. Interestingly, Ank et al. (82) found that HepG2 cells (a human liver carcinoma cell line) showed upregulated expression of IFNL1 and IFNL2/3 upon treatment with either type I or type III IFN. However, in our study we did not detect induction of IFNL1 by those cytokines in A549 cells. These different results could be a consequence of the different cell types used in their study or of limits of detection. While human type I and type III IFNs are known to be induced by direct virus infection by similar transcription factors, their promoters are significantly different. There are 13 subtypes of human IFNA genes, and they are regulated by IRF3 and IRF7, which bind a cluster of transcription sites in the promoters with different affinities (83, 84). However, the regulation of expression of IFNB1 is more tightly regulated by the coordinated binding of multiple complexes of transcription factors, specifically IRF3/IRF7, nuclear factor κB (NF-κB), and activator protein 1 (AP1), to the positive regulatory domains (PRD) of the enhanceosome (85). The type III IFN has been more recently described, given its later discovery (86, 87). Human IFNL2 and IFNL3 are almost identical and have highly similar promoters (86, 87). IFNL4 is expressed in only a fraction of the human population, due to a widespread genetic polymorphism introducing a 5′-proximal frameshift (88). All IFNL promoters have binding sites for NF-κB and for IRF3/7, with some differences among them. In general, the literature suggests that IFNL promoters are more flexible than the type I IFN promoter (8), which agrees with the broader distribution of type III IFN found in our study, and that NF-κB plays an important role in its activation. IFNL1 regulation is very divergent from IFNL2/3 (13). IFNL1 has a distal cluster of NF-κB binding sites upstream of the promoter, and engagement of these sites by NF-κB transcription factors is enough to activate its expression (8, 89). Additionally, IFNL1 promoter can be activated by IRF and NF-κB factors independently through the separated elements, although engagement of both is necessary for the highest levels of expression (89). Therefore, the paracrine induction of IFNL1 could be due to its promoter organization, which is unique among the rest of the type I or type III IFNs. Alternatively, additional binding sites for transcription factors yet unidentified could be present in the IFNL1 promoter, which could contribute to the unexpected paracrine induction of this gene. The broad expression of IFNL1 suggests a possible mechanism of amplification of the antiviral response in IFNLR1-IL10R2-expressing tissues, such as the respiratory epithelium, during IAV infections.

Our study also highlights the appropriateness of single-cell transcriptome analyses to better understand the processes governing virus-host interactions. In this study, this technology allowed for the identification of patterns of expression of IFN genes and ISGs in infected cultures, suggesting that the ability of a cell to secrete IFN and the degree of their antiviral capacity are associated with multiple factors, such as their infection state and the levels of the expression of viral genes. These issues cannot be addressed by the use of bulk analysis. Given the recent development of this type of technology, only a few studies have been reported to date applying them to IAV infection. Two recent *in vitro* studies focused on the heterogeneity of viral transcripts during IAV infection in epithelial cells and on the characterization of virus species triggering the immune response (43, 80). In these studies, very low induction of the expression of innate immune genes was found, probably due to the use of a low MOI, which minimized the presence of coinfections as required by the type of questions addressed. An interesting *in vivo* study also addressed similar questions but focused mostly on the heterogeneity of infection and innate immune responses across multiple cell types (45).

We also find that early innate immune responses developed after IAV infection at low MOIs are efficient at inhibiting subsequent infection of bystander cells. If the virus overcomes this first barrier and propagates in the tissue by multiple viruses infecting the same cells, the virus would more efficiently block the innate immune response in infected cells and hypothetically could replicate more efficiently as infection progresses. Therefore, this report provides insights into how the dynamics of the interplay of IAV immune antagonism and innate immune response can change as the number of viruses infecting single cells increases in the respiratory epithilium after the early stages of infection.

## MATERIALS AND METHODS

### Cell culture.

Human adenocarcinoma A549 and Madin-Darby canine kidney (MDCK) cells were purchased from the ATCC and cultured in Dulbecco’s modified Eagle medium (DMEM, Gibco) and minimal essential medium (MEM; Gibco), respectively, supplemented with 10% fetal bovine serum (FBS; VWR), l-glutamine, and penicillin-streptomycin (Gibco).

Improvement of cell nucleus segmentation for microscopy and image processing was achieved with the generation of A549 cells stably expressing an mCherry fluorescent protein-fused histone H2B (A549 H2B-mCherry cells). The H2B-mCherry expression plasmid was a gift from Robert Benezra (Addgene plasmid number 20972) (90). Human embryonic kidney (HEK) 293T cells were cotransfected with H2B-mCherry plasmid in combination with 3rd-generation lentiviral packaging plasmids pMDLg/pRRE, pRSV-Rev, and pMD2.G (gifts from Didier Trono; Addgene plasmids 12251, 12253, and 12259). The resulting lentiviral particles were used to transduce A549 cells. Single-cell clonal selection was performed, and a selection of monoclonal candidates was tested against wild-type A549 cells to discard phenotypic abnormalities.

Primary normal human bronchial epithelial (NHBE) cells were purchased from Lonza. Progenitor cells were expanded and differentiated as previously described (1). Briefly, cells were expanded in bronchial epithelial cell growth medium (BEGM; Lonza) and then cultured in 12-mm Transwell filters (Corning) for 4 to 6 weeks in a 1:1 BGEM-DMEM mixture (differentiation medium) supplemented with retinoid acid (sigma) at a final concentration of 15 ng/ml.

### Preparation of virus stocks.

IAV strains A/California/4/2009 (H1N1), A/Wyoming/03/2003 (H3N2), and A/Puerto Rico/8/1934 expressing NS1 fused to green fluorescent protein (PR8-GFP) were propagated in specific-pathogen‐free embryonated hens’ eggs (Charles River Laboratories) (91). Infectious titers of IAV stocks were determined by standard plaque assay on MDCK epithelial cells. For infections of A549 cells, infectivity was measured by influenza virus hemagglutinin (HA) staining and automated imaging using a Celigo imaging cytometer (Nexcelom), and the titer was adjusted to obtain approximately the expected infectivity for each multiplicity of infection (MOI) according to the Poisson distribution.

### Infections.

Infections of A549 H2B-mCherry cells for real-time microscopy and single-cell transcriptome sequencing analysis was performed up to 12 h postinfection (hpi) to capture the first replication cycle of IAV. Cells were plated the day before the experiment in 24-well plates. Virus inoculum was prepared in phosphate-buffered saline (PBS), and 100 μl was added to the cells. After 1 h at 37°C, the inoculum was removed and Eagle’s MEM (EMEM; Lonza) supplemented with 5% NaHCO_3_, penicillin-streptomycin (Gibco), l-glutamine (Gibco), and 1% FBS was added to the cells. Cell culture plates were then placed in the 37°C, 5% CO_2_ chamber of the Zeiss LSM 880 Airyscan microscope for subsequent imaging or collected at the desired time points for single-cell RNA transcriptome analysis.

NHBE cells were washed 10 times with PBS prior to infection in order to remove mucins. The virus inoculum was prepared in PBS, and 200 μl (or PBS for mock-infected samples) was added per well. After 45 min, the inoculum was removed, cells were washed with PBS, and the PBS was removed, so the infection progressed in an air-liquid interphase. The infections with the H3N2 virus were done in a 200-μl inoculum in combination with 20 μg/ml of anti-H1 neutralizing antibody 7B2, specific for the head of A/California/4/2009 (52). Cells were incubated in the presence of this mixture for 45 min, and this mixture was subsequently removed. Then cells were washed once with PBS and an additional 30-min incubation with 20 μg/ml of anti-H1 antibody in PBS was performed, after which the antibody was removed and cells were incubated in an air-liquid interphase for the appropriated times. This protocol was tested first to ensure the inhibition of the H1N1 replication. Also, we confirmed that the addition of the 7B2 antibody had no effect on the replication or the innate immune profile induced by the H3N2 virus. A pan-H3 monoclonal antibody, 9H10, was used to determine the titers of H3N2 viruses (92). At the specified time points, 200 μl of PBS was added over the infected cells; PBS was collected after incubation for 15 min at 37°C for subsequent replication assessment by plaque assay. Cells were lysed and kept at −80°C for RNA extraction and qRT-PCR analysis. All the NHBE cell infections were done in triplicate.

### qRT-PCR.

Quantitative reverse transcriptase PCR (qRT-PCR) was performed to measure gene expression levels as previously described (93), with some modifications. Briefly, RNA isolation was performed using the Agencourt RNAdvance Cell v2 kit (Beckman Coulter). cDNA was synthesized from total RNA with AffinityScript Multi-Temp RT (Agilent Technologies). PCR PlatinumTaq DNA polymerase and a SYBR green (Life Technologies)-containing buffer were used for the PCR, which was performed in a thermocycler (ABI7900HT; Applied Biosystems). The RPS11, TUBA1B, and ACTB housekeeping genes were used as internal controls. All samples were run in triplicate and normalized to the median threshold cycle (*C_T_*) value of the three corrected control genes in each sample, with the value converted to a nominal copy number per cell by assuming 2,500 copies of ACTB mRNA molecules per cell and an amplification efficiency of 93% for all reactions. In the NHBE cell experiment, results were then normalized to the 1-hpi time point of its respective time course and log_10_ transformed. Primer sequences can be found in Table 1. Data analysis was performed using R version 3.3.1 and Rstudio.

**TABLE 1:** Primers used for analysis of gene expression by qRT-PCR

Gene	Forward sequence	Reverse sequence
IFNA2	CTTGCCCGTATTTTTAGGAC	TGACAGAGACTCCCCTGATG
IFNB1	GTCAGAGTGGAAATCCTAAG	ACAGCATCTGCTGGTTGAAG
IFNL1	GCCTCCTCACGCGAGACCTC	GGAGTAGGGCTCAGCGCATA
IFNL2	TCTGGAGGCCACCGCTGACA	TGGGCTGAGGCTGGATACAG
IFNL3	TGGCCCTGACGCTGAAGGTT	CGTGGGCTGAGGCTGGATAC
IFIT2	CGTGGGAACCTGGTGACTAA	TCGTTCCAAGCATACCGTGA
IFITM1	CTATGCCTCCACCGCCAAGT	TGTCACAGAGCCGAATACCA
IFITM3	TGGTCCCTGTTCAACACCCTC	CCCTAGACTTCACGGAGTAGGC
CXCL10	TCCCATCACTTCCCTACATG	TGAAGCAGGGTCAGAACATC
IRF7	GGTGTGTCTTCCCTGGATAG	GCTCCAGCTCCATAAGGAAG
ISG15	TGGACAAATGCGACGAACCTC	CTGCGGCCCTTGTTATTCCTC
Mx1	CGTGGTGATTTAGCAGGAAG	TGCAAGGTGGAGCGATTCTG
OAS1	TTTGATGCCCTGGGTCAGTT	GTGCTTGACTAGGCGGATGA
RIG-I	AAAGCCTTGGCATGTTACAC	GGCTTGGGATGTGGTCTACT
CCL5	AAGCTCCTGTGAGGGGTTGA	TTGCCAGGGCTCTGTGACCA
CCL8	GTTAAATCTGGCAACCCTAG	CAACATCACTGTGAGGTAAG
RPS11	GCCGAGACTATCTGCACTAC	ATGTCCAGCCTCAGAACTTC
ACTB	ACTGGAACGGTGAAGGTGAC	GTGGACTTGGGAGAGGACTG
TUBA1B	GCCTGGACCACAAGTTTGAC	TGAAATTCTGGGAGCATGAC

### Time-lapse microscopy.

A Zeiss LSM 880 Airyscan microscope was used for the collection of the PR8-GFP/A549 H2B-mCherry cell time-lapse microscopy images. A total of 25 images per well (in a 24-well plate) were collected every hour between 3 hpi and 12 hpi at a magnification of ×20. Automated quantification of the data was performed using custom-made MatLab scripts. Cells were segmented using the H2B-mCherry nuclear tracker as mask. We optimized the tiling and tile stitching strategies for the image acquisition, background reduction algorithms, and segmentation strategies. Data such as position (*x* and *y* coordinates), median of fluorescence intensity of GFP (normalized to the maximum fluorescence of each well), and infection status were extracted for each cell (>25,000 cells/well). The graph and heat map in Fig. 1 were generated using R v3.3.1 and Rstudio.

### Single-cell RNA sequencing.

A549 H2B-mCherry-infected or mock-infected cells were detached from the cell culture plates using trypsin-0.05% EDTA (Gibco), washed with cell culture media, and passed through a 40-μm cell strainer to prepare single-cell suspensions, and the final concentration was set at 1,000 cells/μl. We performed single-cell encapsulation with beads using the 10× Genomics Single Cell 3′ v2 kit and following manufacturer instructions (10× Genomics, Pleasanton, CA). Briefly, a 10× microfluidic chip was loaded to target 5,000 cells. Single-cell gel beads in emulsion (GEMs) were generated, and reverse transcription was performed in the emulsion prior to 12 cycles of cDNA amplification. Quality control and quantification of the amplified cDNA were assessed by Bioanalyzer. Libraries were constructed according to the manufacturer’s instructions. Each library was tagged with a different index for multiplexing during sequencing. Library quality controls were assessed by Bioanalyzer and quantified by Qubit and quantitative PCR (KAPA Biosystems quantification kit for Illumina platforms). Sequencing was performed on an Illumina HiSeq 2500 (Illumina, Inc., San Diego, CA) using the following read lengths: 26 bp for Read1 (cell barcode plus UMI), 8 bp for i7 index (sample index), and 98 bp for Read2 (insert).

### Data analysis of single-cell RNA sequencing.

The following steps were followed for data preprocessing and analysis. Paired-end reads from Illumina sequencing were aligned to a combination of the human hg19 and viral PR8 genomes using the Cell Ranger (10× Genomics, Pleasanton, CA) pipeline. The resulting unique molecular identifier (UMI) count matrices were automatically filtered to remove background and noncellular barcodes. Six samples representing MOIs of 2, 0.2, and 0 (mock) at 4 h and 12 h were analyzed. The numbers of cells sequenced were 6,758, 7,377, and 4,505 for MOIs of 2, 0.2, and 0, respectively, at 4 hpi and 4,579, 4,698, and 8,562 for MOIs of 2, 0.2, and 0, respectively, at 12 hpi. Over 18,000 distinct genes were detected in each sample.

Sequencing analysis was performed using MATLAB and the single-cell sequencing analysis R package Seurat (94). We filtered cells by requiring a minimum of 200 genes with nonzero UMI counts and filtered genes by requiring a minimum of 3 cells with nonzero UMI counts. Cell UMI counts were then normalized for all samples to 10,000 total UMIs per cell, excluding viral genes, as they undergo a separate transcription procedure within infected cells. We performed principal-component analysis (PCA) to reduce data dimensionality. A graph-based clustering approach was implemented over the first 10 PCA dimensions to separate our cell populations into distinct clusters. Manual curation of these clusters was then performed, with clusters combined to reflect the three relevant cell states we found to be present in our samples: uninfected or bystander cells, infected cells producing high levels of IFN, and infected cells producing low levels of IFN. Differentially expressed genes were determined for each cluster via a nonparametric Wilcoxon rank sum test, with top markers included in heat maps for the samples at 12 h after infection at MOIs of 2.0 and 0.2. To minimize the effect of rare barcode or sequencing alignment errors, we require a raw UMI count greater than 1 for a gene to be considered expressed. Pearson correlation coefficients were calculated between viral genes and cellular genes upregulated by viral infection. Viral and cellular genes were then sorted by their median correlation coefficients against the respective gene sets.

Pathway analysis was performed using the Reactome pathway Database (42), which uses a statistical (hypergeometric distribution) test that determines whether certain Reactome pathways are overrepresented.

### Immunofluorescence assay in NHBE cells.

We evaluated the infectivity of H1N1 IAV in NHBE cells by immunofluorescence. Cells were fixed with 4% paraformaldehyde, permeabilized with 0.1% Triton X-100, and blocked with 10% FBS in PBS. Next, cells were stained with an anti-HA (pan-HA group 1) monoclonal antibody KB2 (39) and a secondary goat-anti-mouse IgG, Alexa Fluor 488 (A488; Invitrogen), as well as a secondary goat anti-mouse IgG, Alexa Fluor 647 (A647; Invitrogen), and 4′,6-diamidino-2-phenylindole (DAPI; Sigma). Images of complete Transwells (in triplicate) were collected using the automated Celigo imaging cytometer (Nexcelom). The percentage of infected cells was calculated by gating positive cells in A488 and A647 to minimize background noise, using the DAPI signal as a mask for nucleus segmentation.

### Cytokine stimulation experiments.

A549 cells were stimulated with recombinant TNF-α (Gibco), IFN beta (PBL Assay Science), IFNL1 (PBL Assay Science), or different combinations of these cytokines for 8 h at 100 U/ml in EMEM supplemented with 5% NaHCO_3_, penicillin-streptomycin (Gibco), l-glutamine (Gibco), and 1% FBS. Also, some cells were infected with PR8-NS1-GFP at an MOI of 2 as a positive control. Cells were lysed and stored at −80°C for subsequent RNA extraction and qRT-PCR analysis.

### Data availability.

RNA sequencing data are available at the GEO repository under accession number GSE122031.
